# Editorial: Shared Decision Making in Mental Health: International Perspectives on Implementation

**DOI:** 10.3389/fpsyt.2021.793284

**Published:** 2021-12-07

**Authors:** Shulamit Ramon, Alan David Quirk, Yaara Zisman-Ilani

**Affiliations:** ^1^Department of Allied Health, Midwifery and Social Work, University of Hertfordshire, Hatfield, United Kingdom; ^2^Royal College of Psychiatrists, London, United Kingdom; ^3^Department of Social and Behavioral Sciences, College of Public Health, Temple University, Philadelphia, PA, United States; ^4^Department of Clinical, Educational and Health Psychology, Division of Psychology and Language Sciences, University College London, London, United Kingdom

**Keywords:** shared decision making, mental health, adults and children, primary care, ethnic minorities

While there is an increased acceptance of the potential usefulness of applying share decision making (SDM) in everyday mental health practice, its implementation in practice is still lacking. People who use mental health services often do not know what SDM is; clinicians often have reservations concerning the capacity of service users to make decisions, and fear that SDM may lead to harmful risk taking in increased medication non-adherence. Current research of these issues demonstrates the importance of easy access to information concerning mental health interventions, and the relevance of respect and trust by both clinicians and service users to each other in the process of SDM. Existing research highlights the willingness and ability of most people experiencing mental ill health to reach well- informed decisions alongside their clinicians.

The editors of this special issue have therefore invited articles on original research describing SDM projects contributing to advancing the development of SDM and its implementation.

Twelve articles were accepted for publication in this special issue.

Two articles addressed the need to adapt SDM to non-western cultures and underserved racial/ethnic minority populations, emphasizing needed adaptations to make SDM accessible to address cultural aspects. Matthews et al. conducted formative qualitative research in the US, to understand SDM perception among underserved ethnic minority patients with depression in primary care. Results point to stigma and lack of trust in the provider and the system as critical barriers for SDM in depression primary care.

Ismail and Midin describe the first SDM study in Malaysia focusing on SDM preferences of adults with schizophrenia. Based on a cross-sectional design, the authors concluded that although the Malaysian with schizophrenia prefers to be involved in SDM, the practice is limited due to providers' lack of interest in developing SDM.

These articles are in line with a growing critique of the recent NICE SDM guidelines Zisman-Ilani et al. (2021), which exclude variations of SDM practices that have been made to address different policies, cultures and health conditions.

Gutman et al. focus on training health and social care undergraduate and postgraduate Israeli students to learn what SDM is and SDM application in practice placements. The study highlights the complexity of the application to practice due to lack of sufficient knowledge of SDM and of its implementation by their practice teachers, as well as the lack of organizational commitment to SDM.

Stanhope et al. from the US describe the development of a 10-item Person-Centered Care Planning Assessment Measure, a measure of the extent to which mental health services are person-centered.

Vitger et al. from Denmark present a systematic review and meta -analysis of digital SDM intervention in mental health. They concluded that digital interventions to support SDM in mental health is promising, but more evidence is needed.

Three articles focus on parents, children, and young adults. In two articles from the UK, Liverpool et al. and Liverpool et al. looked at the underlying emotional layer experienced by parents taking part in SDM concerning their child who has mental health problems, a layer rarely looked at. Based on qualitative analysis, results show that negative emotional states hindered active participation in the SDM process, while a positive such state encourages an active participation.

The second article by the same groups provides a secondary analysis of a large sample concerning the degree of participation by parents in SDM. The findings highlight that parents of Asian origin and parents of children with learning difficulties had a high level of participation, while the presence of conduct problems among children predicted a lower level of SDM.

Simmons et al. describes a first step of qualitative formative development of an online decision aid to empower young people identified as likely to develop psychosis to become active participants in an SDM process concerning their care using focus groups with both clinicians and clients.

Two articles focused on interventions with family members and service users. Ramon looked at the currently existing evidence concerning the development of the application of Family Group Conferences (FGC) to adults with mental health issues, which has originated in social work. The FGC aims to enhance family involvement in supporting these adults, reigniting the willingness of family members to do so in a meaningful way by devising an action plan and participating in its implementation.

Weiss et al. have developed the Rainbow approach in Israel. They applied a structural cognitive modifiability framework to the practice of enabling family members to improve the relationships they have with an adult member experiencing mental ill health, with considerable success. The article focused on the hitherto hidden aspect of SDM necessary for facilitating communication between parents and their adult son or daughter.

Last, two articles focused on SDM in psychiatric medication management. Kaminskiy et al. describe a formative qualitative analysis of services users, nurse prescribers and psychiatrists on barriers and enablers for SDM around antipsychotic medication. A small group of services users acted as co-interviewers and co-researchers.

Fox provided a first-hand perspective of a service user experiencing two different types of involvement in mental health SDM.

The 12 articles published in this special issues of Frontiers Psychiatry offer the following:

- Explore creatively, yet methodically, at least one key issue of SDM in mental health.
- Apply in an evaluated way SDM across a whole mental health service.
- Identify barriers to implementing SDM and attempt systematically and ethically to devise ways of overcoming the barriers.
- Use facilitators to enhance the implementation of SDM in mental health in a systematic way.
- Pay attention to reducing the power differential between service users and clinicians, while considering ways of increasing more equal collaboration within the research design.
- Address SDM with minority populations.
- Focus attention to the development of SDM with different sub-populations and services (e.g. parents and clinicians of children mental health services, families of adult clients, community mental health services, primary care, families of adult clients, young adults).
- Provide international coverage; articles come from Australia, Denmark, Israel, Malaysia, UK and the US.

## Author Contributions

All authors listed have made a substantial, direct, and intellectual contribution to the work and approved it for publication.

## Conflict of Interest

The authors declare that the research was conducted in the absence of any commercial or financial relationships that could be construed as a potential conflict of interest.

## Publisher's Note

All claims expressed in this article are solely those of the authors and do not necessarily represent those of their affiliated organizations, or those of the publisher, the editors and the reviewers. Any product that may be evaluated in this article, or claim that may be made by its manufacturer, is not guaranteed or endorsed by the publisher.

